# RPC Teacher-Based Program for Improving Coping Strategies to Deal with Cyberbullying

**DOI:** 10.3390/ijerph16060948

**Published:** 2019-03-16

**Authors:** Annalisa Guarini, Damiano Menin, Laura Menabò, Antonella Brighi

**Affiliations:** 1 Department of Psychology, University of Bologna, 40127 Bologna, Italy; laura.menabo@studio.unibo.it; 2 Department of Education Studies “Giovanni Maria Bertin”, University of Bologna, 40126 Bologna, Italy; damiano.menin2@unibo.it; 3 Faculty of Education, Free University of Bolzano, 39042 Brixen-Bressanone, Italy; antonella.brighi@unibz.it

**Keywords:** cyberbullying, coping strategies, teacher based-intervention, adolescents

## Abstract

Background: Cyberbullying is a serious threat to public health and teachers can play a key role in its detection, prevention and intervention. The present study evaluated the effectiveness of the RPC (“Relazioni per crescere”—Relationships to Grow) program, a short intervention, implemented at classroom level by trained teachers, aimed at improving awareness on cyberbullying and increasing proactive coping strategies to deal with cyberbullying behaviors. Method: The effectiveness of the RPC project was analyzed through an observational study (pre/post-intervention comparison), involving 898 Italian students of Lower Secondary schools (6th–8th grades). Results: Hierarchical logistic regression showed that after the intervention students were more likely to consider the different roles in cyberbullying (cyberbully, cybervictim, reinforce/assistant, defender and bystander/observer). In addition, hierarchical linear regressions highlighted an improvement of social coping and cognitive coping strategies after the intervention. Conclusions: RPC is a short, teacher-based program that can increase the awareness of cyberbullying among students and improves their effective coping strategies to address cyberbullying. Further research on the efficacy of short teacher-based programs would be worthwhile, given the limited financial and time resources of the schools, emphasizing the active and crucial role of teachers in tackling cyberbullying.

## 1. Introduction

Even if cyberbullying is a recent phenomenon described the first time 15 years ago, it is a widespread problem among students around the world [1] and it constitutes a serious public health issue, since it has been associated with a decrease in wellbeing and an increase in symptoms of depression, anxiety and low self-esteem [2,3,4]. Moreover, several episodes in the last 10 years have pointed out the extreme effects of victimization, such as suicide [5]. In addition, cyberbullying has an impact on the learning environment at school, negatively affecting the school climate [6]. 

At the international level, bullying and cyberbullying are considered forms of psychological and physical violence and they represent a violation of Article 9 of the UN Convention on the Rights of the Child (UNCRC). At the European level, there are no specific legal instruments targeting cyberbullying, but the EU has the role of coordinating and supporting the national initiatives of Country members and to promote Directives on victims’ rights [7]. In Italy, both a law to contrast cyberbullying [8] and guidelines for schools [9] have been recently approved, since previous studies have shown that cyberbullying is a severe phenomenon in Italian schools. Indeed, a higher rate of cyberbullying among Italian students in comparison to Spanish and UK students was described [10,11]. A high incidence of cyberbullying among Italian students was confirmed by another European project, suggesting higher implication in cyberbullying in Poland, Italy and Greece compared to Spain, UK and Germany [12]. In addition, cyberbullying was described as a widespread phenomenon in Italy since similar incidences were found among different regions [13]. Finally, the phenomenon was already diffused (around 10%) among pre-adolescents as described by a large sample representative of the Italian Lower Secondary school population [14]. Starting from these results, the Italian law [8] aimed at preventing and identifying cyberbullying in all its forms. In particular, the Italian law stressed the relevance of, in each school, designating a teacher who can coordinate actions to prevent and contrast cyberbullying. This indication was integrated in the Italian Guidelines of Ministry of Education, Universities and Research [9], that suggest the need to implement a nationwide teacher training campaign in order to empower teachers’ capacity to detect risky online behaviors and cyberbullying phenomena, adopting an interdisciplinary approach.

As acknowledged by the Italian enactments, the role of teacher is very crucial in identifying, preventing and intervening against cyberbullying. However, there is converging evidence that teachers do not perceive themselves to be adequately prepared for this task, suggesting that more research needs to be conducted in order to understand how schools and communities can intervene with cyberbullying [3,8,9,10]. In an Australian study, teachers reported to be less likely to recognize instances of cyberbullying, and were more uncertain about how to address bullying involving technology, compared to other forms of bullying [15]. Fewer than 10% of Australian secondary school staff reported feeling very skilled to deal with cyberbullying, while 50% felt poorly or not at all skilled to do so [16]. Other evidences showed that although teachers were aware they should do more to prevent cyberbullying [17] and they recognized cyberbullying as a problem, their perspectives and ideas on effective strategies to prevent cyberbullying were largely inconsistent and they highlighted the need for training on cyberbullying [15,18,19].

Concerning teacher training, very few interventions entirely delivered by teachers have been described in the literature. An example of a manual-based intervention entitled “Media Heroes” was implemented in Germany [20]. Teachers, after training, proposed several activities in their classes. The long version of the intervention (10 weeks) revealed a reduction of cyberbullying and an increase of affective empathy, while the short version (1 day) of the intervention showed a positive effect of cognitive empathy [20]. The Spanish “Asegúrate Program” was another example of targeted interventions against cyberbullying to be implemented by teachers, taking into account the theory of normative social behavior, self-regulation skills and the ideas/belief held by adolescents. Results showed a decrease in aggression and cyberaggression thanks to the intervention [21]. 

Alongside these specific intervention projects, modules for teachers’ training have been included into “whole school” approaches to tackle bullying and cyberbullying. As an example, the Cyber-Friendly Schools (CFS) project was a comprehensive whole-school intervention against bullying and cyberbullying. In this program teachers implemented one third of the contents [22]. Additionally, the Tabby Improved Prevention and Intervention Program (TIPIP) was a whole school approach combining the Ecological System Theory and the Threat Assessment Approach. A teacher training module was one out of the four components of the project (teachers, parents, in class activities and online materials) and it aimed at describing the cyberbullying phenomenon, risk factors for students’ involvement, how to prevent and manage cyberbullying, and legal issues [23]. The KiVa Antibullying Program consisted of both universal and focused actions with a particular attention to bystanders. A teacher training and a teacher’s guide that provided step-by-step instructions for the curriculum lessons (20 h) were included in the program [24]. The KiVa program, developed to contrast bullying behaviors, has shown a good efficacy in reduction of cybervictimization and cyberbullying [24]. In the NoTrap! Program, a short training module for teachers was implemented too, even if the intervention was carried on through the peer educators’ interventions [25]. 

Besides the specific components included in each intervention package, it should be taken into consideration what the prevention program is aimed at. A meta-analysis by Van Cleemput et al. [26] pointed out that many studies described a qualitative or quantitative evaluation of a prevention program aimed at reducing cyberbullying and cybervictimization among 10 to 18 year olds or at changing its proximal determinants (knowledge, attitudes, social skills, coping strategies). The outcomes of a program, indeed, can differ significantly across the different approaches, with some programs specifically aimed at obtaining a reduction of cyberbullying or cybervictimization rates, while other programs appear to be more focused on reducing the exposure to risk factors for cyberbullying and/or on improving protective factors, such as proactive and functional coping strategies. In this line, the strategy to invest in health promoting behaviors can be more beneficial [26], in light of the consideration that risk-reduction behavior is harder to change than health-promoting behavior, such as increasing appropriate coping skills [27]. 

For this purpose, it might be useful to consider what literature identifies as “effective” and “ineffective” coping strategies. Slee and Murray-Harvey [28] examined experts’ views on the effectiveness of coping strategies utilized by Australian secondary school students and they found that items rated by the experts as “effective” and “ineffective” strongly aligned with the theoretical description of coping proposed by Lazarus [29]. Lazarus differentiated among problem-focused coping strategies (directed toward managing or altering the problem causing distress) and emotion-focused coping strategies (directed at regulating the emotional response). In the study by Slee and Murray-Harvey [28], behaviors like “talk to a school counselor” and “get support from others”, which fall into the category of problem focused coping strategies, were rated by the experts as effective, while statements/actions like “see myself at fault” and “wish for a miracle”, which were expressions of an emotion-focused coping style were generally rated as ineffective by the experts. The strategies adopted to cope with cyberbullying may influence the persistency of victimization [30,31,32], as well as its effect on mental health of the victim. Interventions should be designed to empower students by targeting their attitudes, problem solving skills, and their sense of control and to assist them to respond more effectively to being victimized [33,34].

To our knowledge, very few intervention programs aimed specifically at promoting and evaluating changes in students’ coping strategies to cyberbullying, and none of them was fully delivered by teachers. Pieschl and Urbasik [35] found a significant increase in the use of technical coping styles, but they found no differences in the use of other coping styles (i.e., retaliation, proactive, withdrawal). Lam and Frydenberg [36] did not find significant changes in the use of productive coping styles, non-productive coping styles or seeking social support after the intervention.

The RPC program (“Relazioni per Crescere”, “Relationships to Grow”) was a universal, modularized and theoretically based intervention developed to help teachers in preventing and contrasting cyberbullying in their classes, through the promotion of health-related behaviors and by fostering positive relationships among students. It built on previous knowledge about potential risk and protective factors such as empathy and coping skills. This shift towards the consideration of protective factors besides the risk factors acknowledges the suggestions elaborated in the framework of Developmental Psychopathology [37,38].

The RPC is a short intervention program entirely delivered by teachers (6 h of teacher training; four activities proposed by teachers in their class during school hours; 1.5–2 h for each activity; 1 h of teacher supervision with expert psychologists). The program content is focused on the main following areas that, as suggested by a recent review [4], have been identified as the key components included in effective programs against cyberbullying.

1. Digital literacy. Risky information and communications technology (ICT) use was one of the main predictors of cyberbullying perpetration and cyberbullying victimization, as revealed by a recent meta-analysis [39]. High-risk actions such as sharing passwords, talking to strangers, and uploading intimate information on social networks made victims more vulnerable [40]. In addition, cyberbullying was also in comorbidity with other Internet risks, such as sextortion and online grooming [41].
2. Awareness raising and education on cyberbullying. Students needed to increase their awareness on cyberbullying, particularly for females [42]. Awareness-raising concerning the moral implications and the harm that can be caused to others by content manipulation, offensive language, social exclusion, threats, etc., has proven to be effectively pursued by some programs as ConRed [43]. Awareness raising can also target the social dynamics of bullying and cyberbullying, since many students behave in ways that maintain, even fuel, the bullying behavior. This aspect has been particularly emphasized in the KiVa program [44].
3. Communication and social skills. Card and Hodges [45] found a lack of social skills/competence among the victims of violent bullying, and this may also be mirrored in cyberbullying [40]. Moreover, since cyberbullying arises frequently from face to face interactions at school, improving the social and communicative skills of the class-group could result in better relationships among students, thus also in a long term reduction of cyberbullying [46].
4. Empathy training. The need of empathy training in reducing cyberbullying behavior was highlighted by previous studies [47]. In particular both the cognitive empathy (recognizing and understanding another’s emotional state) and the affective empathy (subjective state from emotional contagion) were inversely associated with cyberbullying [20].
5. Coping skills. Several types of coping strategies were described in relation to cyberbullying: confronting, technical solutions, supportive strategies and avoidant strategies [48]. Coping skills to deal with cyberbullying may exacerbate or may help to reduce the intensity of the aggression and can have significant associations with mental health. In the case of cyberbullying, it appeared that the negative consequences were influenced by the use of ineffective coping strategies, and the use of ineffective coping appeared to keep bullying and cyberbullying going [49]. Therefore, the intervention program aimed at improving proactive problem-focused coping strategies (cognitive or social) and to reduce passive/avoidant strategies.

Based on previous research results, the present paper examined the effects of RPC intervention based at the classroom level and implemented by trained teachers, aimed at improving awareness, and increasing proactive coping strategies. We hypothesized that this short intervention may increase students’ knowledge of the phenomenon and improve students’ coping skills in dealing with cyberbullying. In addition, a possible reduction of cyberbullying and cybervictimization was expected, even if this was not the aim of the intervention since this trend was not found by previous studies which proposed short interventions [20].

## 2. Methods

### 2.1. Participants

In the present study, 898 students filled in a questionnaire before and at the end of intervention. Students were recruited from 35 Public Lower Secondary schools from all the nine provinces of Emilia-Romagna region (North-Centre of Italy). Data of the Emilia-Romagna region revealed that 18% of students did not have an Italian citizenship (for more details see http://istruzioneer.gov.it/dati/fact-sheet/). According to the data of the HBSC Study [50], in the Emilia-Romagna region 64% of the students in the age range from 11 to 15 years lived with two parents. For what concerns the level of education of the parents, in the Emilia-Romagna region the results from the HBSC study [50] revealed that, in the range from 11 to 13 years of age, 31% of mothers had a University degree, 37% an Upper Secondary school degree, 12% a Vocational training qualification, 18% a Lower Secondary school degree, and 2% a Primary school certification. Fathers had lower levels of education compared to mothers: 26% of fathers had a University degree, 35% an Upper Secondary school degree, 13% a Vocational training qualification, 24% a Lower Secondary school degree, and 2% a Primary school certification. Concerning socioeconomic status (SES), the Family Affluence Scale revealed that the population of the Emilia-Romagna region was comprised of 17% in the low level, 53% in the medium level, and 30% in the high level. Data about the general SES of the families in Emilia-Romagna showed that this region reports families with higher levels of SES compared to the medium score of other Italian regions. As established by the Italian Ministry of Education [51], the subjects to be taught in Italian Lower Secondary schools are Italian, two other languages—English plus Spanish, French or German—History, Geography, Math, Science, Music, Arts, Physical Education, Technologies. Preliminary skills of cyber literacy are included in Technologies. 

Concerning the year level of our sample, 198 students attended the 6th grade (22%), 473 the 7th grade (53%), and 227 the 8th grade (25%). The age of participants ranged from 10 to 15 years (M = 12.15, SD = 0.83). The participants are a convenience sample, because the schools decided autonomously to take part to the intervention study and indicated to the research team which classes would have implemented the RPC program. The gender composition of the sample was balanced: 49% were females (*n* = 438) and 51% were males (*n* = 460). The data were collected from 2017 and 2018. No other anti-bullying programs have been carried out in the involved schools. 

### 2.2. Questionnaire

The questionnaire consists of two different sections concerning cyberbullying and coping strategies. 

Cyberbullying was assessed using two 11-item scales from the European Cyberbullying Intervention Project Questionnaire (ECIPQ; [2,12,20]). The questionnaire covered different behaviors including direct and indirect aggression and social exclusion online (e.g., “Someone spread rumors about me online”/“I spread rumors about someone online”, “Someone created a fake account pretending to be me online”/“I created a fake account pretending to be someone else online”). Students were asked to answer to each item on a 5-points Likert scale (0 = never; 1 = one or two times; 2 = monthly; 3 = weekly; 4 = several times in the last week). Cronbach’s alphas were respectively 0.82 and 0.83 for cyberbullying and cybervictimization scales. In addition, an open-ended question was asked in order to investigate the awareness of different roles involved in cyberbullying (“Who do you think is involved in an episode of cyberbullying?”).

Strategies to cope with cyberbullying were assessed using an adapted and translated version of the “Coping with Bullying Questionnaire” [52]. The scale included four subscales, measuring respectively cognitive (10 items, e.g., “Think of different ways I could solve the problem”), social (eight items, e.g., “Ask a teacher for help with the cyberbullying”), passive (five items, e.g., “Wish a miracle would happen to stop the cyberbullying”) and confrontational coping (four items, e.g., “Fight back”). Participants were asked to indicate their responses to real or hypothetical situations on each item on a 5-point Likert scale (from 0 = never to 4 = always). Cronbach’s alphas were respectively 0.76 for cognitive and confrontational coping, 0.87 for social coping and 0.55 for passive coping. The last one was therefore excluded from further analysis since it was not a robust variable.

### 2.3. RPC Program

Teachers attended a training course (6 h) with expert psychologists concerning theoretical definitions of bullying and cyberbullying phenomena and the explanation of activities to carry out with their students. Teachers received also a manual with step-by-step descriptions of the activities and some materials that they could use in their classes. The following in-class activities were proposed by teachers (four activities; 1.5–2 h for each activity):

(1) Digital literacy. Using brainstorming, teachers debated with students’ risks and opportunities of ICTs in order to improve safe use of technologies.
(2) Awareness raising and education on cyberbullying. Starting from different scenarios, students in small groups identified roles involved in cyberbullying and they co-constructed the definition of cyberbullying.
(3) Empathy training. Students experienced different roles in cyberbullying through a short role-playing in order to improve both cognitive empathy (recognizing another’s emotional state) and affective empathy (“How I felt in this role”).
(4) Coping skills. Students in small groups produced slogans to contrast cyberbullying taking into account the different roles involved in the phenomenon (“What can be useful for a victim? What can a bystander do?”). The different types of coping strategies were analyzed, identifying which can be more effective in contrasting cyberbullying.

Communication and social skills were improved in all activities since dialog, discussion and negotiation among students were encouraged. At the end of each activity, students produced materials (posters, slogans, pictures) that allowed to both synthesize the contents and to keep the main messages for the class in the future. The activities were proposed by teachers in their classes within a two-month period. During these months 1 h of supervision of teacher activities was provided by expert psychologists in order to support teachers in the program implementation.

### 2.4. Coding

Participants were categorized into four groups based on cybervictimization and cyberbullying scores for descriptive purposes. Students who admitted to have perpetrated at least one type of online aggression on a monthly basis, or admitted to at least two different types of online aggression were considered as bullies [12]. Students who reported having suffered at least one type of online aggression on a monthly basis, or reported having suffered at least two different types of online aggression were considered as victims [12]. Based on this classification, participants were assigned to one of the four mutually exclusive groups: “Not Involved”, “Pure Victims”, “Pure Bullies” and “Bullies/Victims”.

Average scores were calculated for each scale (cyberbullying, cybervictimization, social, cognitive and confrontational coping). 

The coding of the open-ended question “Who do you think is involved in an episode of cyberbullying?” was performed using CAQDAS software NVivo (version 11, QSR International, Melbourne, Australia). Based on a priori definition of different roles involved in bullying [53], five categories were identified: bully, victim, reinforce/assistant, defender and bystander/outsider. For each category, two expert psychologists selected a set of semantically keywords. 

Answers provided by participants to the open question in pre- and post-intervention were analyzed to quantify the occurrence of each category. Repetitions within the same answer were not considered. In addition, the number of empty answers and “I do not know” answers were counted.

### 2.5. Procedure and Study Design

A repeated measures design was adopted to investigate the effectiveness of the intervention. Students filled in online questionnaires during school hours. The first questionnaire was filled in during the week before the intervention (pre-intervention) and the second questionnaire within two weeks after the end of in-class activities (post-intervention). Teachers remained in the classrooms during the survey in order to clarify any questions or problems. All questionnaires were anonymous, and a nickname was chosen by the student and used for data collections (pre-intervention and post-intervention) in order to match the responses.

### 2.6. Ethics

The study protocol met the ethical guidelines for the protection of human participants, including adherence to the legal requirements of Italy, and received a formal approval by the Bioethics Committee, University of Bologna. Both parents gave their informed written consent for the participation to the study.

### 2.7. Statistical Analysis

Regression analyses were run using the lmerTest package under R version 3.5.2 (R Foundation for Statistical Computing, Vienna, Austria) and the significance level was set at 0.05. Multilevel linear regressions were fitted to inquire the potential changes in average scores for cyberbullying and coping-related variables after the intervention (cyberbullying, cybervictimization, social, cognitive and confrontational coping). The hierarchical structure of the data set was modeled by including a random intercept, with students nested in classes, nested in schools. Gender and age were included as predictors in the regressions in order to control for their potential effects.

Binary variables resulting from the coding of the question “Who do you think is involved in cyberbullying?” were analyzed via hierarchical logistic regressions, in order to assess whether the understanding of cyberbullying social dynamics varied following the intervention.

## 3. Results

### 3.1. Cyberbullying/Cybervictimization

Table 1 displays the distribution of participants across role-based groups. About 30% of participants, both in pre-intervention and post-intervention, were involved in cyberbullying as bully, victim, or bully-victim. 

No significant changes in average scores for cyberbullying or cybervictimization were highlighted by multilevel regressions (Table 1). No gender- or age-related differences were found.

### 3.2. Awareness of Cyberbullying Social Dynamics

As displayed in Table 2, after the intervention the number of students mentioning keywords pertaining to the category victim was doubled (pre-intervention, 28.6%; post-intervention, 63.7%). Similarly, 66% of respondents (*n* = 593) recognized the role of the bully after the intervention, with a relevant increase compared with pre-intervention assessment (39.8%). The roles of reinforce/assistant and bystander/outsider were almost non-existent before the intervention (below 1%), while after the program the role of reinforce/assistant was acknowledged by 5.2% of participants and that of bystander/outsider by 12.9% of respondents. The number of those recognizing the role of defender increased from below 1% before the intervention to 2.2% after the intervention, but its occurrence was deemed too low for regression analyses. The number of those not answering the question decreased from 10.1% before the intervention to 3% after the intervention. 

Hierarchical logistic regressions confirmed differences between the pre- and post-intervention surveys regarding the different roles in cyberbullying. After the intervention, participants were more likely to mention at least one word associated to the category victim, bully, reinforce/assistant, and bystander/outsider compared with the pre-intervention (Table 2). In addition, after the intervention, participants were found to be less likely to either not answer the question or answer “I don’t know”, compared to before the intervention (Table 2).

### 3.3. Coping Strategies

Hierarchical linear regressions highlighted some differences between the pre- and post-intervention surveys regarding coping strategies (see Table 3 for descriptive statistics).

As highlighted in Table 3, the score for social coping was higher in the post-intervention assessment, compared with the pre-intervention assessment. Social coping also displayed higher scores for girls compared to boys, *t* (1642) = 7.51, *β* = 0.12, *p* < 0.001. A post-intervention increase was also found for cognitive coping (Table 3). Cognitive coping also highlighted a gender difference, with girls having higher scores than boys, *t* (4997) = 4.15, *β* = 0.11, *p* < 0.001.

Confrontational coping did not change significantly after the intervention, highlighting only a positive association with age, *t* (1673) = 2.21, *β* = 0.07, *p* = 0.028.

## 4. Discussion

The present study evaluated the effectiveness of the RPC program, a short intervention, implemented at the classroom level by trained teachers, aimed at improving awareness of cyberbullying and increasing proactive coping strategies in dealing with cyberbullying. We also analyzed a possible reduction of cyberbullying and cybervictimization behaviors.

Concerning the awareness of cyberbullying, the RPC program increased students’ knowledge of the social dynamic of cyberbullying, making them more aware of the interplay among the different roles involved in this phenomenon. Indeed, at the open question “Who do you think is involved in an episode of cyberbullying?” students showed to identify, after in-class activities, the different roles involved in cyberbullying and they were more aware that cyberbullying was a group phenomenon characterized by the following roles: cyberbully, cybervictim, reinforce and assistant, defenders and bystanders/outsiders [53]. The awareness that cyberbullying was a group process and a social phenomenon [53], pointed out the important role of bystanders: this was the first step to promote responsibility in the class and to change the normative rules that may also support and foster aggressive behaviors online [44]. 

The RPC program also showed a significant improvement of students’ coping skills in dealing with cyberbullying. Coping refers to “conscious efforts individuals use to regulate emotion, cognition, behavior, internal states, or situation to reduce threat” [54]. The coping process starts with threat appraisal, i.e., the perceptions of how stressful the event is for the individual. According to Transactional Model of Stress and Coping [55], appraisal happens at two levels: one is the primary appraisal, which assesses the situation in order to determine whether it is a threat; the secondary appraisal assesses the changeability of the situation (or the possibility to change it) along with the individual’s resources to manage the associated stress. These cognitive appraisals determine the coping style selected. In light of our results, where an increase of the problem-focused strategies emerged after the intervention, we may hypothesize that a better knowledge of the cyberbullying phenomenon may have influenced the coping strategies chosen to deal with it. Indeed, a clearer picture of the social dynamics implied in this phenomenon, as shown by the qualitative analysis of the different roles involved in cyberbullying, may have facilitated the two levels of the appraisal process: (a) by providing the students with information helpful to detect the malicious intention behind certain messages; (b) by providing them with a set of resources to deal with it, including those available in the school environment (i.e., peers, teachers, the school policy). This may have led students to indicate more effective coping strategies as the ones we have reported: asking for help to friends, teachers and parents (knowing that there are teachers prepared to support), thinking about how to solve the situation. These strategies showed to be more available for girls than for males before the intervention (as reported in the literature [56]), but increased for both the genders after the intervention. The adoption of assertive coping, such as the confrontational one, was not influenced significantly by the intervention. This latter emphasizes the victim’s attempt to assume an active position with respect to the bully. Specifically, this strategy involves seeking a direct but not aggressive confrontation with the bully in order to stop his/her behavior [57], and it is widely used in cases where the source of the bullying is known [58,59], and in less serious cases of online harassment [60]. It would be relevant to explore the use of this coping strategy in relation to the intensity of the cyberbullying suffered by the victims.

Concerning the effect of the RPC program on the reduction of cyberbullying and cybervictimization, we found that the prevalence of the phenomenon was stable between pre- and post-intervention. Our results revealed that a short intervention (8 h in class), as the RPC program, can improve the awareness of the phenomenon and increase the use of effective coping strategies, but it did not have an effect on the reduction of rates of cyberbullying. Our findings were in line with the results of the short Media Heroes intervention [20]. Indeed, while the long-term (10 weeks) “Media Heroes” program was effective in reducing cyberbullying and promoting affective empathy, the short Media Heroes (1 day, four sessions of 90 min) program was not effective in reducing cyberbullying, reporting a significant effect only on cognitive empathy. Other programs targeted for teachers that described an effect on the reduction of cyberbullying were more extensive (eight sessions for the “Asegúrate Program” [21]). In addition, the RPC program cannot be compared to other programs using a “whole school approach” ([22,23,24]). These programs, in fact, even if they were effective in reducing cyberbullying [61], were expensive and required high support from the schools as well as a great time investment. Unfortunately, as suggested by educators, even if some programs were promising, time constraints, lack of colleagues’ support and uncooperative parents could limit their effect [62]. For this reason, a short program, as RPC, could be a valid tool to increase the awareness of the cyberbullying phenomenon and to promote effective coping strategies. The promising results, however, suggest the efficacy of the RPC programme should be analyzed in a long-term follow-up; if the results obtained in such a short intervention would remain stable in a longer period, this would improve its cost/benefit ratio.

### Limitations

The first limitation was the study design. An observational study was carried out with an analysis of change between pre- and post-intervention phases. The absence of a control group did not allow the investigation of both the effect of maturation and the role of increased awareness. Several studies revealed that cyberbullying increased in control groups, suggesting that without an intervention the phenomenon could grow [20]. Other studies suggested that the intervention modified the awareness, attitude and self-perceptions in dealing with bullying and cyberbullying [63]. Further studies with the RPC program should be carried out using a quasi-experimental design with a control group, since, as suggested by a recent review [4] research at a school does allow true randomization. The collection of a control group could help to understand the effect of the RPC program on cybervictimization and perpetration, taking into account the effect of maturation and the role of awareness. 

A second limitation of the present study was that only the global effect of the intervention was analyzed, without disentangling the effects of specific components of the interventions. As suggested by a recent review and meta-analysis [61] this is a very important challenge, since even if we know that prevention and intervention programs for cyberbullying are effective, we need to explain which specific components of the intervention are more relevant. 

The third limitation concerned the implementation of the intervention: dosage and fidelity. Even if all students involved in the present research took part into the four in-class activities, we did not know if the teachers used all the materials proposed and followed the suggestions from the manual. A more complex index of implementation should be added in further research, since individual and interpersonal factors are important for successful implementation of the program [44]. 

Measures of SES background were not collected directly in the present study. More detailed information would be useful in further studies to improve the generalization of our findings and for understanding the role of these variables in moderating the effect of intervention.

The last limitation of the present study was the selective focus on cyberbullying behaviors. We decided this selective focus following the indication of Italian law [8]. We also needed to implement a short program that could have been suitable for teachers during their curricular hours (Reading/Spelling, Maths, etc.), since no extra hours were available for intervention projects. However, since the cyberbullying phenomenon has comorbidities with other behaviors such as bullying and sextortion [64], we can hypothesize that programs also including activities to contrast these phenomena, could be more effective in reducing cyberbullying [24]. In addition, the lack of specific questions about sextortion and online sexual victimization could have contributed to underestimating the cybervictimization, mainly among females [65].

## 5. Conclusions

RPC is a short and friendly program targeted at teachers that increases the awareness on cyberbullying among students and improves their effective coping strategies to address cyberbullying. Programs like RPC should be part of the schools’ daily activities proposed by teachers. Teacher-based and short interventions can sometimes be the only possibility to trigger schools’ initiatives to prevent cyberbullying, since this kind of intervention may fit with the limited financial and time resources of the schools. In addition, it may respond, at least in Italy, to the recommendations enacted by the Ministry of Education concerning the active involvement of teachers in actions against cyberbullying.

## Figures and Tables

**Table 1:** Cyberbullying and cybervictimization before and after the intervention.

Cyberbullying	Pre-Intervention	Post-Intervention	*t*	*β*	*p*
Not Involved	601 (71.1%)	589 (70.1%)			
Pure Victims	124(14.7%)	128 (15.2%)			
Pure Bullies	43 (5.1%)	38 (4.5%)			
Bullies-Victims	77 (9.1%)	85 (10.1%)			
Cyberbullying	1.07 (0.17)	1.07 (0.20)	0.40	0.01	0.689
Cybervictimization	1.13 (0.29)	1.14 (0.30)	0.97	0.02	0.331

Note: No. (percentage) for role-based groups (percentage was calculated excluding missing values; 53 cases in the pre-intervention assessment and 58 cases in the post-intervention assessment); mean (SD) for cyberbullying and cybervictimization scores.

**Table 2:** Awareness of cyberbullying social dynamics before and after the intervention.

Roles	Pre-Intervention		Post-Intervention		Wald	*b*	*p*
	Yes	No	Yes	No			
Victim	257 (28.6%)	641 (71.4%)	572 (63.7%)	326 (36.3%)	13.01	2.08	<0.001
Bully	357 (39.8%)	541 (60.2%)	593 (66.0%)	305 (34.0%)	10.86	1.42	<0.001
Reinforce/Assistant	5 (0.6%)	893 (99.4%)	47 (5.2%)	851 (94.8%)	10.18	8.82	<0.001
Bystander/Outsider	7 (0.8%)	891 (99.2%)	116 (12.9%)	782 (87.1%)	13.81	10.83	<0.001
Defender	4 (0.4%)	894 (99.6%)	20 (2.2%)	878 (97.8%)			
Missing/No answer	91 (10.1%)	807 (89.9%)	27 (3.0%)	871 (97.0%)	−8.81	−4.85	<0.001

Note: Yes: No. (percentage) of the participants who mentioned at least once one of the words included in the respective category.

**Table 3:** Coping strategies before and after the intervention.

Coping Strategies	Pre-Intervention	Post-Intervention	*t*	*β*	*p*
Social Coping	1.70 (1.08)	1.89 (1.06)	5.11	0.10	<0.001
Cognitive Coping	1.47 (0.77)	1.58 (0.74)	3.55	0.07	<0.001
Confrontational Coping	1.89 (1.18)	1.98 (1.12)	1.45	0.03	0.147

Note: Mean (SD) for each category.

## References

[1] Zych I., Ortega-Ruiz R., Del Rey R. (2015). Systematic review of theoretical studies on bullying and cyberbullying: Facts, knowledge, prevention, and intervention. Aggress. Viol. Behav..

[2] Brighi A., Melotti G., Guarini A., Genta M.L., Ortega R., Mora-Merchán J., Smith P.K., Thompson F., Li Q., Cross D., Smith P.K. (2012). Self-Esteem and Loneliness in Relation to Cyberbullying in Three European Countries. Cyberbullying in the Global Playground.

[3] Espelage D.L., Hong J.S. (2017). Cyberbullying Prevention and Intervention Efforts: Current Knowledge and Future Directions. Can. J. Psychiatry.

[4] Hutson E., Kelly S., Militello L.K. (2018). Systematic Review of Cyberbullying Interventions for Youth and Parents With Implications for Evidence-Based Practice: Cyberbullying Interventions for Individual Youth and Parents. Worldviews Evid.-Based Nurs..

[5] Hinduja S., Patchin J.W. (2010). Bullying, Cyberbullying, and Suicide. Arch. Suicide Res..

[6] Hinduja S., Patchin J.W. (2011). Cyberbullying: A Review of the Legal Issues Facing Educators. Prev. School Fail. Altern. Educ. Child. Youth.

[7] European Union Policy Department C: Citizens’ Rights and Constitutional Affairs. Cyberbullying among Young People. http://www.europarl.europa.eu/RegData/etudes/STUD/2016/571367/IPOL_STU(2016)571367_EN.pdf.

[8] Italian Cyberbullying Law N. 71/2017 (2017). Disposizioni a tutela dei minori per la prevenzione ed il contrasto del fenomeno del cyberbullismo.

[9] MIUR (Italian Ministry of Education, University and Research) Aggiornamento Linee di Orientamento per la prevenzione e il contrasto del cyberbullismo.

[10] Genta M.L., Smith P.K., Ortega-Ruiz R., Brighi A., Guarini A., Thompson F., Tippett N., Mora-Merchàn J., Calmaestra J., Li Q., Smith C.D. (2012). Comparative Aspect of Cyberbullying in Italy, England and Spain: Findings From a DAPHNE Project. Cyberbullying in the Global Playground. Research from International Perspectives.

[11] Ortega R., Elipe P., Mora-Merchán J.A., Genta M.L., Brighi A., Guarini A., Smith P.K., Thompson F., Tippett N. (2012). The Emotional Impact of Bullying and Cyberbullying on Victims: A European Cross-National Study: Emotional Impact of Bullying and Cyberbullying. Aggress. Behav..

[12] Del Rey R., Casas J.A., Ortega-Ruiz R., Schultze-Krumbholz A., Scheithauer H., Smith P., Thompson F., Barkoukis V., Tsorbatzoudis H., Brighi A. (2015). Structural validation and cross-cultural robustness of the European Cyberbullying Intervention Project Questionnaire. Comput. Hum. Behav..

[13] Brighi A., Guarini A., Palermiti A.L., Bartolo M.G., Genta M.L. (2011). Victimization in traditional bullying and cyberbullying among Italian preadolescents. An investigation in Emilia Romagna, Tuscany and Calabria. Età Evolutiva.

[14] Vieno A., Gini G., Lenzi M., Pozzoli T., Canale N., Santinello M. (2015). Cybervictimization and somatic and psychological symptoms among Italian middle school students. Eur. J. Public Health.

[15] Cross D., Shaw T., Hearn L., Epstein M., Monks H., Lester L., Thomas L. (2009). Australian Covert. Bullying Prevalence Study (ACBPS).

[16] Barnes A., Cross D., Lester L., Hearn L., Epstein M., Monks H. (2012). The Invisibility of Covert Bullying Among Students: Challenges for School Intervention. Aust. J. Guid. Couns..

[17] Green V.A., Johnston M., Mattioni L., Prior T., Harcourt S., Lynch T. (2017). Who is responsible for addressing cyberbullying? Perspectives from teachers and senior managers. Int. J. School & Educ. Psychol..

[18] DeSmet A., Aelterman N., Bastiaensens S., Van Cleemput K., Poels K., Vandebosch H., Cardon G., De Bourdeaudhuij I. (2015). Secondary school educators’ perceptions and practices in handling cyberbullying among adolescents: A cluster analysis. Comput. Educ..

[19] Macaulay P.J.R., Betts L.R., Stiller J., Kellezi B. (2018). Perceptions and responses towards cyberbullying: A systematic review of teachers in the education system. Aggress. Viol. Behav..

[20] Schultze-Krumbholz A., Schultze M., Zagorscak P., Wölfer R., Scheithauer H. (2016). Feeling cybervictims’ pain: The Effect of Empathy Training on Cyberbullying. Aggress. Behav..

[21] Del-Rey-Alamillo R., Mora-Merchán J.A., Casas J.-A., Ortega-Ruiz R., Elipe P. (2018). “Asegúrate” Program: Effects on cyber-aggression and its risk factors. Comunicar.

[22] Cross D., Shaw T., Hadwen K., Cardoso P., Slee P., Roberts C., Thomas L., Barnes A. (2016). Longitudinal impact of the Cyber Friendly Schools program on adolescents’ cyberbullying behavior: Impact of the Cyber Friendly Schools Program. Aggress. Behav..

[23] Sorrentino A., Baldry A., Farrington D. (2018). The Efficacy of the Tabby Improved Prevention and Intervention Program in Reducing Cyberbullying and Cybervictimization among Students. Int. J. Environ. Res. Public Health.

[24] Williford A., Elledge L.C., Boulton A.J., DePaolis K.J., Little T.D., Salmivalli C. (2013). Effects of the KiVa Antibullying Program on Cyberbullying and Cybervictimization Frequency Among Finnish Youth. J. Clin. Child Adolesc. Psychol..

[25] Palladino B.E., Nocentini A., Menesini E. (2016). Evidence-based intervention against bullying and cyberbullying: Evaluation of the NoTrap! program in two independent trials: Evaluation of the NoTrap! Program. Aggress. Behav..

[26] Van Cleemput K., DeSmet A., Vandebosch H., Bastiaensens S. A systematic review and meta-analysis of the efficacy of cyberbullying prevention programs. Presented at the Etmaal van de Communicatiewetenschap.

[27] Adriaanse M.A., Vinkers C.D.W., De Ridder D.T.D., Hox J.J., De Wit J.B.F. (2011). Do implementation intentions help to eat a healthy diet? A systematic review and meta-analysis of the empirical evidence. Appetite.

[28] Slee P. Murray-Harvey R Experts’ views on students’ strategies for copying with bullying. Presented at the ISRA XV111 World Meeting.

[29] Lazarus R.S. (1999). Hope: An Emotion and a Vital Coping Resource Against Despair. Soc. Res..

[30] Kochenderfer B.J., Ladd G.W. (1997). Victimized children’s responses to peers’ aggression: Behaviors associated with reduced versus continued victimization. Dev. Psychopathol..

[31] Smith P.K., Talamelli L., Cowie H., Naylor P., Chauhan P. (2004). Profiles of non-victims, escaped victims, continuing victims and new victims of school bullying. Br. J. Educ. Psychol..

[32] Kanetsuna T., Smith P.K., Morita Y. (2006). Coping with bullying at school: Children’s recommended strategies and attitudes to school-based interventions in England and Japan. Aggress. Behav..

[33] Terranova A.M. (2009). Factors that Influence Children’s Responses to Peer Victimization. Child Youth Care Forum.

[34] Skrzypiec G., Slee P., Murray-Harvey R., Pereira B. (2011). School bullying by one or more ways: Does it matter and how do students cope?. School Psychol. Int..

[35] Pieschl S., Urbasik S. (2013). Does the cyber bullying prevention program surf-fair work? An evaluation study. From Cyberbullying to Cyber Safety: Issue and Approach in Educational Context.

[36] Lam C.W.C., Frydenberg E. (2009). Coping in the Cyberworld: Program Implementation and Evaluation—A Pilot Project. Aust. J. Guid. Couns..

[37] Mohaupt S. (2009). Review Article: Resilience and Social Exclusion. Soc. Policy Soc..

[38] Rutter M. (2012). Resilience as a dynamic concept. Dev. Psychopathol..

[39] Chen L., Ho S.S., Lwin M.O. (2017). A meta-analysis of factors predicting cyberbullying perpetration and victimization: From the social cognitive and media effects approach. New Media Soc..

[40] Gradinger P., Strohmeier D., Schiller E.M., Stefanek E., Spiel C. (2012). Cyber-victimization and popularity in early adolescence: Stability and predictive associations. Eur. J. Dev. Psychol..

[41] Machimbarrena J.M., Calvete E., Fernández-González L., Álvarez-Bardón A., Álvarez-Fernández L., González-Cabrera J. (2018). Internet Risks: An Overview of Victimization in Cyberbullying, Cyber Dating Abuse, Sexting, Online Grooming and Problematic Internet Use. Int. J. Environ. Res. Public Health.

[42] Elçi A., Seçkin Z. (2019). Cyberbullying Awareness for Mitigating Consequences in Higher Education. J. Interpers. Viol..

[43] Del-Rey-Alamillo R., Casas J.-A., Ortega-Ruiz R. (2012). The ConRed Program, an Evidence-based Practice. Comunicar.

[44] Haataja A., Ahtola A., Poskiparta E., Salmivalli C. (2015). A process view on implementing an antibullying curriculum: How teachers differ and what explains the variation. School Psychol. Q..

[45] Card N.A., Isaacs J., Hodges E.V.E., Miller T.W. (2008). Multiple Contextual Levels of Risk for Peer Victimization: A Review with Implications for Prevention and Intervention Efforts. School Violence and Primary Prevention.

[46] Ortega-Ruiz R. (2012). Knowing, Building and Living Together on Internet and Social Networks: The ConRed Cyberbullying Prevention Program. Soc. Netw..

[47] Ang R.P., Goh D.H. (2010). Cyberbullying Among Adolescents: The Role of Affective and Cognitive Empathy, and Gender. Child Psychiatry Hum. Dev..

[48] Perren S., Corcoran L., Cowie H., Dehue F., Garcia D., Guckin C.M., Sevcikova A., Tsatsou P., Völlink T. (2012). Tackling Cyberbullying: Review of Empirical Evidence Regarding Successful Responses by Students, Parents, and Schools. Int. J. Confl. Viol..

[49] Jacobs N.C.L., Dehue F., Völlink T., Lechner L. (2014). Determinants of adolescents’ ineffective and improved coping with cyberbullying: A Delphi study. J. Adolesc..

[50] HBSC, Health Behavior in School-Aged Children (2016). Stili di vita e salute degli adolescenti. I risultati della sorveglianza HBSC Italia 2014. Regione Emilia-Romagna. https://salute.regione.emilia-romagna.it/documentazione/rapporti/rapporto-stili-di-vita-e-salute-degli-adolescenti-i-risultati-della-sorveglianza-hbsc-2014-in-emilia-romagna-2016/at_download/file/HBSC_RER_2016.pdf.

[51] MIUR (Italian Ministry of Education, University and Research) Decreto 16 novembre 2012, 254. http://www.gazzettaufficiale.it/eli/id/2013/02/05/13G00034/sg.

[52] Murray-Harvey R., Skrzypiec G., Slee P.T. (2012). Effective and Ineffective Coping With Bullying Strategies as Assessed by Informed Professionals and Their Use by Victimised Students. Aust. J. Guid. Couns..

[53] Salmivalli C., Lagerspetz K., Björkqvist K., Österman K., Kaukiainen A. (1996). Bullying as a group process: Participant roles and their relations to social status within the group. Aggress. Behav..

[54] Raskauskas J., Huynh A. (2015). The process of coping with cyberbullying: A systematic review. Aggress. Viol. Behav..

[55] Lazarus R.S., Folkman S. (1985). Coping and adaptation. Handbook of Behavioral Medicine.

[56] Frisén A., Berne S., Marin L. (2014). Swedish pupils’ suggested coping strategies if cyberbullied: Differences related to age and gender. Scand. J. Psychol..

[57] Aricak T., Siyahhan S., Uzunhasanoglu A., Saribeyoglu S., Ciplak S., Yilmaz N., Memmedov C. (2008). Cyberbullying among Turkish Adolescents. CyberPsychol. Behav..

[58] Huang Y., Chou C. (2010). An analysis of multiple factors of cyberbullying among junior high school students in Taiwan. Comput. Hum. Behav..

[59] Price M., Dalgleish J. (2010). Cyberbullying Experiences, impacts and coping strategies as described by Australian young people. Youth Stud. Aust..

[60] Machackova H., Dedkova L., Mezulanikova K. (2015). Brief report: The bystander effect in cyberbullying incidents. J. Adolesc..

[61] Gaffney H., Farrington D.P., Espelage D.L., Ttofi M.M. (2018). Are cyberbullying intervention and prevention programs effective? A systematic and meta-analytical review. Aggress. Viol. Behav..

[62] Cunningham C.E., Rimas H., Mielko S., Mapp C., Cunningham L., Buchanan D., Vaillancourt T., Chen Y., Deal K., Marcus M. (2016). What Limits the Effectiveness of Antibullying Programs? A Thematic Analysis of the Perspective of Teachers. J. School Viol..

[63] Merrell K.W., Gueldner B.A., Ross W.S. (2008). How Effective Are School Bullying Intervention Programs? A Meta-Analysis of Intervention Research. School Psychol. Q..

[64] Wolak J., Finkelhor D., Walsh W., Treitman L. (2018). Sextortion of Minors: Characteristics and Dynamics. J. Adolesc. Health.

[65] Zetterström Dahlqvist H., Gillander Gådin K. (2018). Online sexual victimization in youth: Predictors and cross-sectional associations with depressive symptoms. Eur. J. Public Health.

